# An Update on the Pathogenic Role of Macrophages in Adult-Onset Still's Disease and Its Implication in Clinical Manifestations and Novel Therapeutics

**DOI:** 10.1155/2021/8998358

**Published:** 2021-06-20

**Authors:** Po-Ku Chen, Der-Yuan Chen

**Affiliations:** ^1^Rheumatology and Immunology Center, China Medical University Hospital, Taichung, Taiwan; ^2^College of Medicine, China Medical University, Taichung, Taiwan; ^3^Translational Medicine Laboratory, Rheumatology and Immunology Center, Taichung, Taiwan; ^4^Ph.D. Program in Translational Medicine and Rong Hsing Research Center for Translational Medicine, National Chung Hsing University, Taichung, Taiwan

## Abstract

Increasing evidence indicates a pivotal role of macrophages in innate immunity, which contributes to the pathogenesis of adult-onset Still's disease (AOSD). Despite the available reviews that summarized the pathogenic role of proinflammatory cytokines in AOSD, a systematic approach focusing on the crucial role of macrophages in this disease is still lacking. This review summarizes the updated functions of macrophages in AOSD and their implication in clinical manifestations and therapeutics. We searched the MEDLINE database using the PubMed interface and reviewed the English-language literature as of 31 March 2021, from 1971 to 2021. We focus on the existing evidence on the pathogenic role of macrophages in AOSD and its implication in clinical characteristics and novel therapeutics. AOSD is an autoinflammatory disease mainly driven by the innate immune response. Among the innate immune responses, macrophage activation is a hallmark of AOSD pathogenesis. The pattern recognition receptors (PRRs) on macrophages recognize pathogen-associated molecular patterns and damage-associated molecular patterns and subsequently cause overproduction of proinflammatory cytokines and recruit adaptive immunity. Some biomarkers, such as ferritin and gasdermin D, reflecting macrophage activation were elevated and correlated with AOSD activity. Given that macrophage activation with the overproduction of proinflammatory cytokines plays a pathogenic role in AOSD, these inflammatory mediators would be the therapeutic targets. Accordingly, the inhibitors to interleukin- (IL-) 1, IL-6, and IL-18 have been shown to be effective in AOSD treatment. Gaining insights into the pathogenic role of macrophages in AOSD can aid in identifying disease biomarkers and therapeutic agents for this disease.

## 1. Introduction

Adult-onset Still's disease (AOSD) is a systemic inflammatory disorder characterized by fever, rash, arthritis, liver dysfunction, lymphadenopathy, variable multisystemic involvement, hyperferritinemia, and even life-threatening complications such as macrophage activation syndrome (MAS) [1–4]. AOSD is a rare but important cause of fever of unknown origin [5]. The reported incidence rates of AOSD were 0.16, 0.22, and 0.4 per 100,000 persons in west France [6], Japan [7], and northern Norway [8], respectively. It is considered an autoinflammatory disease (AID) due to its characteristic phenotypes and the absence of detectable autoantibodies [9]. The innate immune system encompasses the germline-encoded pattern recognition receptors (PRRs), including Toll-like receptors (TLRs) and cytosol-expressed nucleotide-binding oligomerization- (NOD-) like receptors (NLRs) [10], which may drive autoinflammation with unknown etiology. Increasing evidence indicates a pivotal role of macrophage activation in the innate immune response with subsequent inflammatory reactions [11], giving rise to the clinical manifestations of AOSD. Moreover, proinflammatory cytokines such as interleukin- (IL-) 1*β*, IL-6, IL-18, and tumor necrosis factor- (TNF-) *α* play a pathogenic role in AOSD [12–18], leading to an implication of new targeted therapies [19–22]. Therefore, the biologics targeting IL-1, IL-6, or IL-18 have been proven effective in the treatment of AOSD [23–28].

With increasing evidence indicating an immunopathogenesis of AOSD, which is attributable to significant advances in using therapeutic targets for AOSD, this review is aimed at summarizing the current research results regarding the pathogenic role of macrophage activation in AOSD and its clinical implication in clinical characteristics and therapeutics.

## 2. Materials and Methods

### 2.1. Literature Search

The present review focuses on the existing evidence on the pathogenic role of the macrophage activation and cytokine storm in AOSD and its clinical implication in therapeutics. We searched the MEDLINE database using the PubMed interface and reviewed the English-language literature as of 31 March 2021, from 1971 to 2021. The search keywords for this updated review included macrophage, innate immunity, immune response, inflammation, pathogenesis, trigger factors, pathogen-associated molecular patterns (PAMPs), damage-associated molecular patterns (DAMPs), TLRs, inflammasomes, proinflammatory cytokines, cytokine storm, MAS, clinical manifestations, AOSD, autoinflammatory disorders, clinical implication, disease activity, and therapeutic strategies. The relevant drugs include corticosteroids, nonsteroidal anti-inflammatory drugs (NSAIDs), conventional synthetic disease-modifying antirheumatic drugs (csDMARDs), biologic DMARDs (bDMARDs), and targeted synthetic DMARDs (tsDMARDs), mainly Janus kinase (JAK) inhibitors.

### 2.2. Study Selection

Two authors (PK Chen and DY Chen) independently assessed the titles and abstracts of articles identified by the literature search and retrieved the relevant full-text articles. Both authors also evaluated the full-text articles for eligibility and examined the selected articles' references for reference. We selected articles if they (1) were probably relevant to the pathogenic role of macrophages or macrophage-derived cytokines in AOSD and (2) were potentially relevant to therapeutic agents targeting macrophage-related cytokines in AOSD. Both authors extracted data from these studies electronically. Our emphasis is on the updated role of macrophages in the pathogenesis of AOSD and the clinical implication in therapeutics by targeting the mediators involved in AOSD pathogenesis.

## 3. Results

### 3.1. Roles of Macrophage Activation in the Innate Immune Responses

The innate immune system provides an early defense to protect the host from invading foreign pathogens, endogenous danger signals, and allergens [29]. The cells (monocytes, macrophages, neutrophils, natural killer cells, and dendritic cells) of innate immunity play a crucial role in maintaining immune homeostasis by recognizing and removing pathogens. These cells interact with the adaptive immune system through cytotoxic reaction or production of antigen-specific antibodies and cytokines [30]. By the real-time imaging platform, Kapellos et al. revealed that bone marrow-derived macrophage priming with Th2 cytokines such as IL-4 and IL-10 resulted in higher phagocytic function compared with M1 polarization [31]. Macrophages promote tissue homeostasis through regulatory and repair functions [32] and could be divided into classically activated macrophages, wound healing or tissue repairing macrophages, and regulatory macrophages based on three different homeostatic activities [33]. Host-derived DAMPs released from damaged tissue, dying cells, or pathogen infections can be recognized by PRRs on macrophages and subsequently initiate an immune reaction [30, 33–34]. TLRs are well known as a type of PRRs that mediate PAMP and DAMP recognition. Upon PAMP and DAMP recognition, TLRs recruit adapter molecules such as myeloid differentiation primary response 88 (MyD88), activate the downstream signal cascade through NF-*κ*B, and drive proinflammatory cytokine expression [35]. The NLRs are a family of intracellular sensors to mediate innate immunity and inflammation. NLRP (nucleotide-binding oligomerization domain, leucine-rich repeat, and pyrin domain) can form multimeric protein complexes in response to stimuli. The assembly of NLRP inflammasomes triggers cascade-1 activation to convert pro-IL-1*β* and pro-IL-18 into mature IL-18 [36–37]. NLRP inflammasomes can be activated by PAMPs such as microbial toxins and whole pathogens, including bacterial, viral, and fungal [38]. They can also recognize danger molecules such as ATP, extracellular glucose, crystals of monosodium urate, and calcium oxalate crystals [39–42]. These observations suggest that the macrophages can be activated through the recognition of various PAMPs and DAMPs by different types of PRRs.

### 3.2. Pathogenic Role of Innate Immunity in AOSD

#### 3.2.1. Triggering Factors of Innate Immunity in AOSD

The exact etiology of AOSD is not fully understood, although various infections, mainly viral infections, have been suggested as possible causative agents [43]. The reported infectious triggers, so-called PAMPs, include cytomegalovirus (CMV), parvovirus B19, Epstein-Barr virus, rubella virus, *Measles morbillivirus*, hepatitis virus, influenza virus, adenovirus, human immunodeficiency virus, *Mycoplasma pneumonia*, and severe acute respiratory syndrome coronavirus 2 (SARS-CoV-2) emerging in late 2019 [43–55]. We demonstrated that parvovirus B19 nonstructural protein (NS)1 might induce IL-1*β* and IL-18 expression by activating NLRP3 inflammasomes in AOSD [56]. Jia et al. recently revealed that CMV DNA was found in the plasma of AOSD patients with new-onset disease or relapses, and CMV infection is strongly associated with the initiation/amplification of inflammation in AOSD [57]. Besides, Bamidis et al. reported a patient who suffered from sequelae of COVID-19 manifested as severe AOSD [55]. In consideration of infectious triggers, innate immunity plays a crucial role in AOSD pathogenesis.

The DAMPs including advanced glycation end products (AGEs), high mobility group box-1 (HMGB1), soluble CD163 (sCD163), macrophage migration inhibitory factor (MIF), and neutrophil extracellular trap (NET) have been implicated in AOSD pathogenesis [1–2, 11, 58]. Accumulating evidence demonstrates a pathogenic role of advanced glycation end products (AGEs) in inflammation [59–60]. Chen et al. revealed that the AGE levels were elevated and correlated with activity scores and ferritin levels in AOSD patients [61], suggesting the involvement of AGEs in AOSD pathogenesis. HMGB1, a member of DAMPs, is released into the extracellular space from macrophages following inflammasome activation [62]. HMGB1 interacts with TLR2, TLR4, or the receptor for AGEs (RAGE) and mediates inflammatory response [63]. Jung et al. demonstrated that elevated HMGB1 levels were correlated with systemic scores and C-reactive protein (CRP) in AOSD patients and associated with skin rash and sore throat [64]. The sCD163, a heme receptor expressed on macrophages, is elevated and related to hyperferritinemia in AOSD patients [65]. MIF, a T lymphocyte-derived cytokine, inhibits random migration of macrophages [66–67] and reduces anti-inflammatory actions of corticosteroids [68]. Increasing evidence indicates that MIF is a proinflammatory cytokine that can upregulate the expression of proinflammatory mediators, including IL-1*β*, IL-2, IL-6, IL-8, TNF-*α*, IFN-*γ*, and prostaglandin E2 [69]. Serum MIF levels were elevated and correlated with disease activity in AOSD patients [70–71]. Zou et al. also revealed highly increased intracellular MIF in monocytes [70], suggesting that macrophages are activated in AOSD and supporting that AOSD is a disease of histiocyte-macrophage system activation [72–73]. Hu et al. showed that NET DNA from AOSD patients exerted a potent capacity to accelerate the activation of macrophages and increased the expression of IL-1*β*, IL-6, and TNF-*α* [74]. In summary, PAMPs or DAMPs can trigger an interplay between host genetic factors and macrophage activation, contributing to AOSD pathogenesis [1–2, 11, 58].

#### 3.2.2. The Common Features of Macrophage Activation in COVID-19 and AOSD

In response to COVID-19 infection, macrophages may be activated and produce proinflammatory cytokines, resulting in the development of systemic hyperinflammation, the so-called cytokine storm [75–76]. A variety of proinflammatory cytokines, such as IL-1*β*, IL-6, IL-8, and IFN-*γ*, were elevated in severe COVID-19 patients [77] and active AOSD patients [12–19], suggesting a common link of the cytokine storm in the pathogenesis of both diseases. Although Meng et al. recently revealed higher IL-6 and IL-10 in severe COVID-19 than in AOSD [78], a clear distinction of cytokine profiles between severe COVID-19 and active AOSD is challenging and needs to be explored in future studies.

#### 3.2.3. Activated Macrophage-Related Mediators as the Disease Activity Indicators in AOSD

PAMPs or DAMPs initiate macrophage activation through PRRs, including TLRs, NLRP3 inflammasomes [79–82], and C-type lectin domain family 5-member A (CLEC5A)/DAP12 complex, and subsequently cause the release of proinflammatory cytokines and activate an adaptive immune response [29, 83]. Virus sensing can trigger TLRs or activate the NLRP3 inflammasome, leading to inflammatory responses in AOSD [56, 84]. Hsieh et al. also revealed elevated expression of NLRP3 inflammasome signaling molecules, which was correlated with disease activity in AOSD patients [85]. Chen et al. demonstrated that the levels of CLEC5A-expressing monocytes were increased and correlated with disease activity and levels of IL-1*β* and IL-18 in AOSD patients [86].

It is well known that ferritin is a characteristic mediator of AOSD [1–3]. The activated macrophages can stimulate the release of ferritin, and elevated H-ferritin expressions in the lymph nodes and skin were correlated with the severity of AOSD [87–88]. Beyond its iron storage role, ferritin takes a pathogenic role in inflammation [89]. The synthesis of ferritin can be upregulated in response to inflammatory cytokines such as IL-1*β* and IL-6. Moreover, ferritin can stimulate inflammatory pathways to amplify the inflammatory process, supporting a hypothesis that ferritin may not only act as a bystander of acute-phase reaction [90]. Ferritin could be exported through the gasdermin D pole [91], and full-length gasdermin D is cleaved into the N-terminal p30 fragment upon activation of inflammasomes. The p30 fragment forms a pore in the cell membrane, through which the activated IL-1*β* and IL-18 are exported from the cell [92]. Recently, Nagai et al. showed that adults or children with Still's disease had elevated serum gasdermin D N-terminal levels correlated with ferritin and IL-18 [93]. Furthermore, the gasdermin D inhibitor could reduce the release of pyroptosis-mediated ferritin by macrophages. In summary, increased ferritin from macrophage activation was correlated with disease activity of AOSD and might serve as an activity indicator of this disease [94].

#### 3.2.4. Inflammatory Reactions and the Related Manifestations of AOSD

Sustained macrophage activation may lead to tissue inflammation with increased secretion of proinflammatory cytokines. After NLRP3 inflammasome activation, caspase enzymes induce the overproduction of IL-1*β* and IL-18, the hallmark cytokines of active AOSD [12–14, 16]. IL-1*β* and IL-18 further promote the secretion of proinflammatory cytokines, including IL-6, IL-8, IL-17A, and tumor necrosis factor- (TNF-) *α* [95–96]. IL-1*β* can also activate macrophages that play a crucial role in the cytokine storm or MAS [97–98]. In the skin, IL-18 is produced in keratinocytes, Langerhans cells, and dermal dendritic cells and may be related to the cutaneous manifestation of AOSD [99]. The locally activated macrophages in the liver produce a high amount of IL-18 and contribute to AOSD-related hepatitis [13, 100]. With this unique feature, IL-18 is the first identified diagnostic marker and indicator of disease activity for AOSD [14, 101].

Chemokines such as IL-8 are produced mainly by activated macrophages and act as the chemotactic agents of inflammatory cells. Chen et al. revealed that the serum IL-8 level was a significant predictor of persistent arthritis [13]. Furthermore, IFN-*γ*-induced chemokines such as C-X-C motif chemokine 9 (CXCL9), CXCL10, and CXCL11 may contribute to inflammatory responses and cutaneous manifestations in AOSD [102]. IL-6 also enhances immune response and inflammatory reactions and contributes to AOSD pathogenesis [19–20, 103]. As a proinflammatory cytokine, IL-6 may be responsible for fever and skin rash, as well as the production of acute-phase proteins in AOSD [13, 104]. Therefore, biologics targeting IL-6 or its receptor have been proved to be effective in the treatment of AOSD.

MAS or hemophagocytic lymphohistiocytosis (HLH) is characterized by excessive macrophage activation accompanied by the cytokine storm, hemophagocytosis, and hyperferritinemia [105]. The possible trigger factors of MAS include infections, medications used, and uncontrolled AOSD [106–108], and it is associated with high mortality in AOSD [109]. Besides, di Benedetto et al. reported that ferritin levels could be used to predict the emergence of MAS in AOSD patients [110], and AOSD and MAS were both considered hyperferritinemic syndrome [111]. Inflammasome-derived IL-18/IL-1*β* were suggested to play important roles in MAS-associated rheumatic diseases [112]. AOSD patients having higher IL-18 levels were more likely to develop MAS, and their IL-18 and ferritin levels were further increased at the time of MAS [113].

### 3.3. Development of New Targeted Therapies

Because AOSD is a rare disease with a heterogeneity of the clinical course, there is currently no concise consensus for treating AOSD. Although corticosteroids and csDMARDs are the standard-of-care treatment for AOSD [22], a significant proportion of patients showed poor therapeutic response or corticosteroid dependence [21, 114]. Given the pathogenic role of proinflammatory cytokines in AOSD, these inflammatory mediators would become the therapeutic targets.

#### 3.3.1. Anti-IL-1 Therapy

Given that IL-1 is implicated in the pathogenesis of AOSD [115–116] and its ligands and receptors are secreted mainly by activated macrophages, the administration of IL-1-blocking agents in AOSD patients seems to be a logical therapeutic approach with a corticosteroid-sparing effect [24–25, 117–120]. The IL-1-blocking agents include anakinra (an IL-1R antagonist), rilonacept (a soluble IL-1 trap molecule), and canakinumab (anti-IL-1*β* monoclonal antibody). The response to anakinra therapy was rapid and sustained in most patients with AOSD [24–25, 117–118]. An open-label randomized study showed that anakinra induced more beneficial responses than DMARDs in corticosteroid-refractory AOSD patients [120]. A meta-analysis revealed that anakinra was effective in treating AOSD with a steroid-sparing effect [121]. Recently, Vastert et al. demonstrated that the use of anakinra could minimize the steroid dose and improve clinical outcomes in children or adults with Still's disease [122]. A systematic review indicated that anakinra treatment was associated with a steroid-sparing effect, and a large proportion of patients could discontinue the use of steroids [123]. A high-dose anakinra has also been successfully used to treat refractory AOSD complicated with life-threatening MAS [124–125]. Rilonacept, an inhibitor of both IL-1*α* and IL-1*β*, has a longer half-life than anakinra. Limited reports revealed that rilonacept effectively treated AOSD patients with the systemic or articular subtype [126–127]. Although a randomized controlled trial was terminated prematurely with the primary endpoint not achieved, canakinumab treatment improved several outcome measures in AOSD [128]. Based on the evidence and consensus, Italian experts recommended that anti-IL-1 therapy was considered relatively safe and effective in treating refractory AOSD patients, especially the systemic subtype patients, as either the first line or a subsequent line of biological treatment [129].

#### 3.3.2. Anti-IL-6 Therapy

IL-6, a pleiotropic cytokine, binds to IL-6R and a 130 kDa signal-transducing *β*-receptor subunit (gp130) forms a functioning hexametric structure [130]. The activation of gp130 induces the phosphorylation of the signal transducer and activator of transcription 1 (STAT1), STAT3, and mitogen-activated protein kinase (MAPK) cascade and then activates proinflammatory reactions [131]. The pathogenic role of IL-6 [12–13, 103] is substantiated by the successful treatment with IL-6-blocking agents in AOSD. The IL-6 receptor antagonist, tocilizumab (TCZ), has recently been proposed as a promising biological agent for AOSD patients. In a case series of 14 patients with intractable AOSD, TCZ therapy resulted in complete resolution of the clinical disease activity in 57% of patients and markedly reduced the maintenance dose of corticosteroids [20]. TCZ is effective in treating AOSD patients with either the systemic or chronic articular patterns [132], including those who were refractory to anakinra [133–135] or TNF-*α* inhibitors [136–137]. Furthermore, TCZ treatment was effective for AOSD patients complicated with MAS [138]. However, macrophage activation syndrome developed following TCZ therapy in one patient with refractory AOSD, implying that caution should be exercised in the very active status of this disease [139]. Based on the previous findings [132–138, 140–141], TCZ treatment is effective and well tolerated in treating refractory AOSD patients.

#### 3.3.3. Anti-IL-17 Therapy

Given the pathogenic role of IL-17 in AOSD pathogenesis [18], the administration of IL-17 inhibitors in AOSD patients seems to be a logical therapeutic approach with a corticosteroid-sparing effect. The IL-17 inhibitors have recently been proposed as a promising biological agent for rheumatic patients [142–143]. Clinical trials showed that anti-IL-17 antibodies significantly reduced rheumatoid arthritis (RA) signs and symptoms and C-reactive protein levels [144–145]. Several monoclonal antibody-mediated IL-17 inhibition approaches for patients with inflammatory diseases have proceeded to phase III clinical trials.

#### 3.3.4. Anti-IL-18 Therapy

IL-18, one member of the IL-1 family, is expressed on monocytes, macrophages, and dendritic cells [146]. The binding of IL-18 to its receptors (IL-18R*α* and IL-18R*β*) triggers proinflammatory reactions. Previous studies revealed that IL-18 levels were elevated and correlated with disease activity in AOSD [12–14], and markedly increased IL-18 levels were reported in AOSD patients complicated with MAS [112]. Given that IL-18 binding protein (IL-18BP) is an inhibitor of IL-18, a phase II clinical trial demonstrated that IL-18BP (Tadekinig alfa) was effective and well tolerated in treating AOSD [28]. Recently, Tadekinig alfa has been shown to have therapeutic effects with a rapid decrease of disease activity in active AOSD patients who were refractory to csDMARDs [147]. These available results indicate that IL-18 may be a promising therapeutic target in AOSD.

#### 3.3.5. Anti-TNF-*α* Therapy

TNF-*α*, an important proinflammatory cytokine, has been reported to be elevated in sera and synovial membranes of AOSD patients compared with osteoarthritis patients or healthy subjects [13, 148]. Although Kraetsch et al. revealed significant improvement in the clinical and laboratory outcomes in 6 AOSD patients receiving infliximab therapy [149], a recent evidence-based review showed that TNF-*α* inhibitors might not be effective in AOSD treatment [137].

#### 3.3.6. Anti-IFN-*γ* Therapy

Given a pathogenic role of interferons such as IFN-*γ* in AOSD [15], the IFN-*γ* blockade may effectively treat AOSD with or without concomitant MAS [150]. Recently, Gabr et al. reported that emapalumab, an IFN-*γ* blockade, effectively eliminated fever and improved laboratory outcomes of a patient with AOSD complicated by MAS [151]. Data regarding the effectiveness of the IFN-*γ* blockade in treating AOSD remain limited.

#### 3.3.7. Janus Kinase (JAK) Inhibitors

Given that JAK inhibitors can block multicytokines, the use of JAK inhibitors may be feasible for AOSD treatment. Kacar et al. reported that baricitinib, a JAK1/2 inhibitor, was effective in treating two AOSD patients who were refractory to csDMARDs and biological therapy [152]. The combination of baricitinib and anakinra therapy effectively treated a patient with refractory AOSD [153]. A recent report from China revealed the successful use of tofacitinib, a JAK1/3 inhibitor, in 14 patients with AOSD [154]. Besides, tofacitinib therapy was effective in treating a patient with AOSD complicated by MAS [155].

## 4. Conclusions

**Figure 1:**
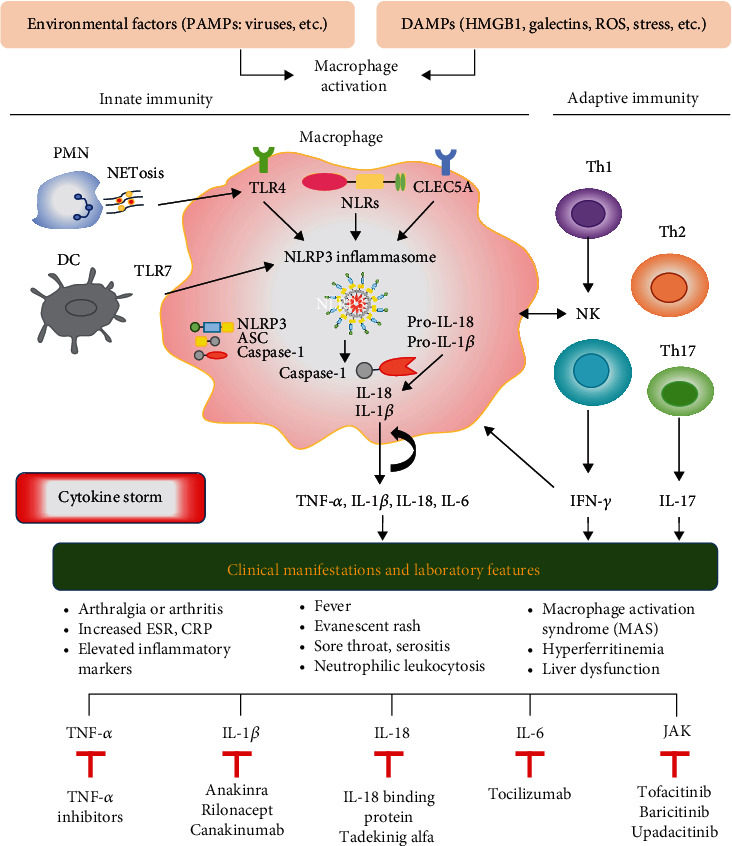
The proposed model for the summary of the pathogenic role of macrophages in adult-onset Still's disease and its implication in clinical manifestations and therapeutics. PAMPs: pathogen-associated molecular patterns; DAMPs: damage-associated molecular patterns; HMGB1: high mobility group box-1; ROS: reactive oxygen species; PMN: polymorphonuclear neutrophils; NETs: neutrophil extracellular traps; DC: dendritic cells; TLRs: Toll-like receptors; NLRs: cytosol-expressed nucleotide-binding oligomerization- (NOD-) like receptors; NLRP: nucleotide-binding oligomerization domain, leucine-rich repeat, and pyrin domain; CLEC5A: C-type lectin domain family 5-member A; Th: helper T cells; NK: natural killer cells; IL: interleukin; TNF: tumor necrosis factor; IFN: interferon.

The status of hyperinflammation in AOSD, mainly driven by an innate immune response, is characterized by an overproduction of proinflammatory cytokines [1–2, 11, 58]. PAMPs or DAMPs initiate macrophage activation through PRRs and subsequently activate adaptive immune responses [29, 83]. The elevated levels of activated macrophage-related mediators may contribute to the clinical manifestations of AOSD and act as the potential therapeutic targets [156]. Accordingly, the inhibitors to IL-1, IL-6, and IL-18 have been shown to be effective in AOSD treatment. The use of TNF-*α* inhibitors, such as infliximab, was effective for AOSD patients with the chronic articular subtype. Through the multicytokine blockade, JAK inhibitors were also an effective treatment for AOSD with or without concomitant MAS. Better insights into the pathogenic role of macrophages in AOSD can aid in identifying disease biomarkers and novel therapeutics. Based on the available evidence of the pivotal role of macrophage activation in AOSD pathogenesis and its clinical implication, we summarized the data as in Figure 1.

## Data Availability

The data supporting the results cited in the text can be found in the relevant articles cited in the references.
